# Immune Tissue Print and Immune Capture-PCR for Diagnosis and Detection of *Candidatus* Liberibacter Asiaticus

**DOI:** 10.1038/srep46467

**Published:** 2017-04-18

**Authors:** Fang Ding, Cristina Paul, Ron Brlansky, John S. Hartung

**Affiliations:** 1 College of Plant Science and Technology, Huazhong Agricultural University, Wuhan, P. R. China; 2 USDA ARS Molecular Plant Pathology Laboratory, Beltsville, Maryland, United States of America; 3 University of Florida, Citrus Research and Education Center, Lake Alfred, Florida, United States of America

## Abstract

‘*Candidatus* Liberibacter asiaticus’ (CaLas), associated with citrus Huanglongbing (HLB), is a non culturable member of the α-proteobacteria. In this study serologically based methods for the detection of CaLas were developed. An anti-outer membrane protein A (OmpA) polyclonal antibody previously produced (in our laboratory) was highly effective for the detection of CaLas from citrus tissues in a simple tissue printing format. The antibody was also used to capture bacteria from periwinkle extracts. About 80% of all field samples analyzed tested positive with both immune tissue printing and qPCR; whereas 95% were positive with at least one of these two methods. When asymptomatic citrus tissues were tested, the tissue printing method gave a higher rate of detection (83%) than the qPCR method (64%). This is consistent with a lower concentration of CaLas DNA, but a higher proportion of viable cells, in the asymptomatic tissues. The immune tissue printing method also highlights the detail of the spatial distribution of ‘*Ca*. Liberibacter asiaticus’ in diseased citrus tissues. Both the immune capture PCR and immune tissue printing methods offer the advantages of low cost, high throughput, ease of scaling for multiple samples and simplicity over current PCR-based methods for the detection of ‘*Ca*. Liberibacter asiaticus’.

Citrus huanglongbing (HLB), also known as citrus greening, is one of the most devastating diseases of citrus and threatens the citrus industry worldwide^1^,^2^,^3^, leading to large reductions in fruit production and quality as well as decline of infected trees. The disease is associated with a phloem-limited and fastidious member of the α-proteobacteriacea, ‘*Candidatus* Liberibacter asiaticus’ (CaLas). CaLas originated in Asia^1^, and is the only species of Liberibacter associated with citrus with a global distribution and is the one associated with HLB disease in the United States. CaLas is a member of the *Rhizobiaceae*, and as is typical for intracellular pathogens, has a significantly reduced genome (1.23 Mb)^4^,^5^. Because the bacterium has not been cultured and classically characterized the name has only ‘*Candidatus*’ status. Typical disease symptoms include yellow shoots within a sector of the tree, with leaves that mature to an asymetric blotchy mottle that may appear to be similar to zinc deficiency. The infected fruit are reduced in size and become lopsided due to seed infection and abortion and off flavors develop^6^,^7^. Vein corking, a symptom also observed with some strains of *Citrus tristeza virus*, was recently reported especially in old citrus leaves^7^. A closely related species, ‘*Ca*. Liberibacter solanacearum’ (CaLso), was recently reported as the causal agent of potato zebra chip disease^8^. CaLso can also infect other hosts like tomato, pepper^9^ and carrot^10^.

Although HLB has been known for more than a century, the fact that CaLas cannot be cultured made it impossible to use traditional bacteriological methods to identify the pathogen and delayed the establishment of the etiology of the disease. In recent years, HLB associated with CaLas has spread widely within China, has become endemic in both Brazil and Florida^7^, and is also present in Texas^11^ and California^12^. Owing to inadequate disease and vector control methods, and the extreme difficulty in protecting citrus from infection by CaLas, commercial citrus industries have suffered great economic losses from HLB and continue to decline^2^. Growers in Florida currently maintain strict control programs for the citrus psyllid vector, even after trees become infected with CaLas to prevent new infections from occurring^13^, and support the tree’s increased nutritional needs by supplemental nutritional programs^14^.

Confirmatory diagnosis of symptomatic and asymptomatic trees remains challenging. For most of the 20^th^ century, the diagnosis of HLB relied on bio-indexing with plants such as *Citrus reticulata* Blanco (mandarin orange) and *Citrus sinensis* (L.) (sweet orange) or the experimental non-rutaceous host, *Catharanthus roseus* (Madagascar periwinkle). Other methods developed to detect and diagnose HLB include Polymerase Chain Reaction (PCR)^15^,^16^,^17^, qPCR^18^,^19^, and Loop Mediated Isothermal Amplification (LAMP)^20^,^21^ and a lateral flow dipstick assay^22^. All of the PCR-based methods require purification of DNA before the assay, which adds to the cost of the assays. CaLas is found only in the sieve tube elements of infected plants^1^,^23^. Although infections are systemic from the roots to the young shoots, the distribution of CaLas is very uneven and on average the concentrations of the pathogen are low in tissues sampled^24^,^25^. Ultrastructural studies have shown that adjacent phloem cells can be completely filled with CaLas or empty^26^,^27^. Furthermore, the population levels of CaLas in individual trees as estimated by qPCR is not well correlated with foliar symptoms^24^. This could be due to the fact that populations of CaLas increase in root tissues long before foliar symptoms become obvious^28^. The mean CaLas concentration in asymptomatic leaves was significantly lower than that in symptomatic leaves as estimated by qPCR^29^.

Serological assays are widely used to diagnose plant diseases, but have not been widely used for HLB because the pathogen has not been available in culture to produce antibodies against CaLas cells. However, proteins produced by CaLas are available for use as antigens by PCR-based cloning and either poly- or monoclonal antibodies can be made against them since the genome of CaLas became available^4^. Due to the limitations of current assays, and the large numbers of trees that must be sampled in citrus production areas where the disease is either present or feared, it is important to develop fast, efficient and inexpensive methods to accurately detect CaLas. Previously, we constructed and produced a highly specific anti-OmpA polyclonal antibody against CaLas^30^,^31^. Here we report the optimization of a simple immune tissue print and demonstrate an immune capture-PCR (IC-PCR) assay based on a polyclonal antibody (Pab) raised in rabbit against the major outer membrane protein (OmpA) of CaLas. These optimized immune tissue print and IC-PCR methods complement existing PCR-based methods and will meet the urgent need for large scale detection of CaLas for the continued sustainability of the United States citrus industry.

## Results

### Optimization of the working dilutions of the anti-OmpA Pab and the goat anti-rabbit conjugated Pab

The dilutions of the anti-OmpA Pab and the secondary goat anti-rabbit Pab were optimized. In a preliminary trial, the anti-OmpA Pab produced a very strong color reaction localized in the phloem cells when a 1:500 dilution was used. Serial dilutions of anti-OmpA Pab, from 1:1,000 to 1:10,000 were then tested with the dilution of goat anti-rabbit secondary Pab held constant at 1:50,000 dilution. When the anti-OmpA Pab was diluted from 1:1,000 to 1:4,000, very strong signals were produced in leaf midrib sections from both CaLas-infected and healthy controls (Fig. S1a–d). Color was observed not only in the phloem cells, but also outside of the phloem tissues. When the anti-OmpA Pab was diluted to 1:5,000 and 1:6,000 (Fig. S1e and f), the difference between diseased and healthy control petiole sections was very pronounced: very strong purple colored spots were seen in the CaLas infected phloem cells, but only a weak pink background was present in the healthy controls. When the anti-OmpA Pab was diluted from 1:7,000 to 1:10,000 (Fig. S1g–j), the purple color was strong in the phloem cells, with very little background color in the healthy controls.

In order to optimize the concentration of the secondary goat anti-rabbit Pab, serial dilutions were made from 1:10,000 to 1:100,000 with the anti-OmpA Pab held constant at a dilution of 1:5,000. A strong purple color was present in both CaLas-infected and healthy control petiole sections when the goat anti-rabbit secondary Pab was diluted from 1:10,000 to 1:40,000 (Fig. S2a–d). When the goat anti-rabbit polyclonal antibody was diluted 1:50,000, a strong purple color was observed in CaLas-infected phloem cells, but there was a weak background in the healthy control (Fig. S2e). As the goat anti-rabbit antibody was diluted further, from 1:60,000 to 1:100,000 (Fig. S2f–j), the intensity of the purple colored spots in the phloem cells was reduced and no purple colored spots were produced in the healthy control.

### Detection of CaLas in different citrus tissues by tissue printing

**Figure 1:**
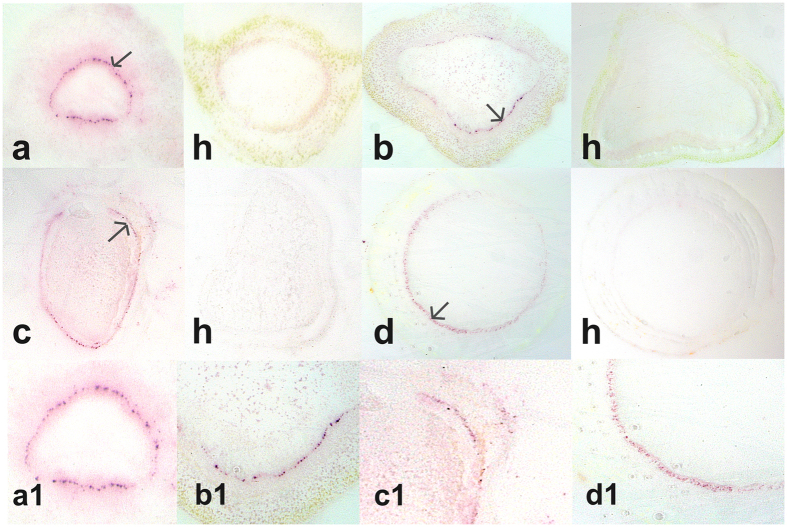
Detection of ‘*Ca*. Liberibacter asiaticus’ in different tissues of sweet orange using tissue prints. (**a**–**d**) Sections of petiole, stem, seed and root; respectively. ‘*Ca*. Liberibacter asiaticus’ in sweet orange is on the left in each panel with healthy sweet orange (**h**) on the right. (a1–d1) Higher magnification of the portions of the images indicated by arrows in **a–d** shows the localization of the purple spots in the phloem cells.

Using the optimized dilutions of both the primary Pab (1:5000) and secondary Pab (1:50,000), petioles, stems, seeds and roots collected from sweet orange were tested. In tissue prints made from the petioles and stems, strong purple colored spots were produced in the phloem cells of CaLas-infected samples, but not in similar tissue prints from healthy controls (Fig. 1a,b). In tissue prints from infected seeds, CaLas was distributed in the seed coat. Tissue prints from seeds obtained from fruit known to be free of CaLas did not produce any purple color (Fig. 1c). In tissue prints prepared from primary roots, we usually observed less purple color in the phloem cells (Fig. 1d) as compared to the phloem cells of petioles and stems. No purple color was observed in tissue prints of roots from healthy controls. When observed with higher magnification, the purple colored spots were sharply focused in individual phloem cells (Fig. 1a1–d1).

### Detection of CaLas by IC-PCR and qPCR from citrus and periwinkle samples

**Figure 2:**
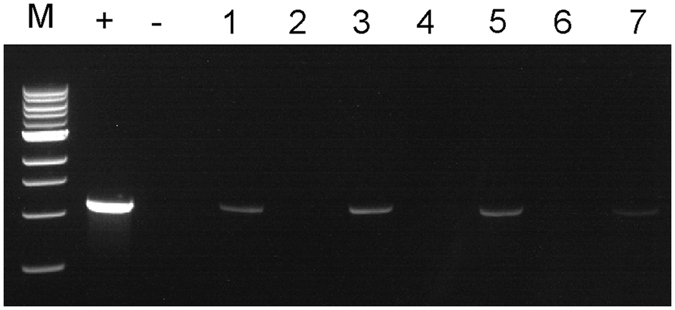
Immune capture-PCR for the detection of ‘*Ca*. Liberibacter asiaticus’ isolate B432 (Florida). M: 1 kb DNA Ladder, +: Purified CaLas DNA positive control; -: water only control. Lane 1, B432 extract and anti-OmpA Pab (1:100); Lane 2, Healthy control with anti-OmpA Pab (1:100); Lane 3, B432 extract and anti-OmpA Pab (1:200); Lane 4, Healthy control with anti- OmpA Pab (1:200); Lane 5, B432 extract and anti-OmpA Pab (1:250); Lane 6, Healthy control with anti- OmpA Pab (1:250); Lane 7, No anti-OmpA Pab was added.

**Figure 3:**
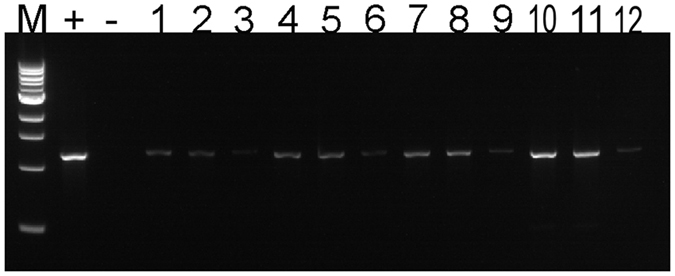
Immune capture-PCR for the detection of ‘*Ca*. Liberibacter asiaticus’ in periwinkle. M: 1 kb Marker.+: Purified CaLas DNA positive control. -: water only control. Crude extract of periwinkle captured in 0.2 ml PCR tubes pre-coated with anti-OmpA Pab at a dilution of 1:200 and sample volumes of 20 μl, 50 μl, 100 μl and 200 μl in lanes 1,2; 4,5; 7,8; and 10,11 respectively. Crude extract of periwinkle captured in 0.2 ml PCR tubes without pre-coating with anti-Omp A Pab and sample volumes of 20 μl, 50 μl, 100 μl and 200 μl in lanes 3, 6, 9 and 12, respectively. Anti-OmpA Pab was used at a 1:200 dilution throughout.

**Figure 4:**
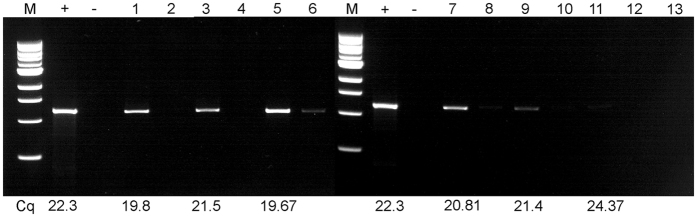
Immune capture-PCR for the detection of ‘*Ca*. Liberibacter asiaticus’ in periwinkle and citrus samples. M: 1 kb Marker.+: Purified CaLas DNA positive control; −: water only control. Lanes 1, 3, 5 and 7: Periwinkle plants DF1, DF2, DF3 and DF4, respectively with anti-OmpA Pab (1:200 dilution); 2, 4, 6 and 8: Periwinkle DF1, DF2, DF3 and DF4 respectively, without anti-OmpA polyclonal antibody; 9. Sweet orange CaLas B232 (Thailand) with anti-OmpA Pab (1:200 dilution); 10. CaLas B232 (Thailand) without anti-Omp A Pab; 11. Sweet orange B430 (Japan) with anti-Omp A Pab (1:200 dilution); 12: B430 without anti-OmpA Pab; 13. Healthy sweet orange control.

IC-PCR was performed to capture CaLas strain B432 (Florida) from infected sweet orange and periwinkle extracts. When PCR tubes were pre-coated with anti-OmpA Pab at dilutions of 1:100, 1:200, 1:250 to capture CaLas from citrus petioles, more PCR amplification product was seen after electrophoresis (Fig. 2 Lanes 1,3,5) than when no anti-OmpA Pab was added to PCR tubes (Fig. 2 Lane 7). Amplification products were not produced from healthy control samples (Fig. 2 Lanes 2,4,6). For the IC-PCR tests of periwinkle extracts, tubes were pre-coated with the anti-OmpA Pab at 1:200 dilution. When different amounts of crude extracts were tested, sample volumes of 100 μl or 200 μl gave much stronger amplification products than when only 20 μl or 50 μl of extracts were used (Fig. 3). When PCR tubes were not pre-coated with anti-Omp Pab as a control, only weak amplification product bands were produced by CaLas-infected samples (Fig. 3 Lanes 3,6,9,12). Detection of CaLas from different periwinkle and citrus isolates using the IC-PCR method was compared with results of DNA extraction followed by qPCR. Samples with lower Cq values when assayed by qPCR generally produced much stronger amplification products when assayed by IC-PCR than did samples with higher Cq values (Fig. 4). The limit of detection for IC-PCR was somewhere near a Cq of 25, because CaLas were not captured and amplified when Cq values were above that point.

### Detection of CaLas in symptomatic and asymptomatic field samples by tissue print and qPCR

**Figure 5:**
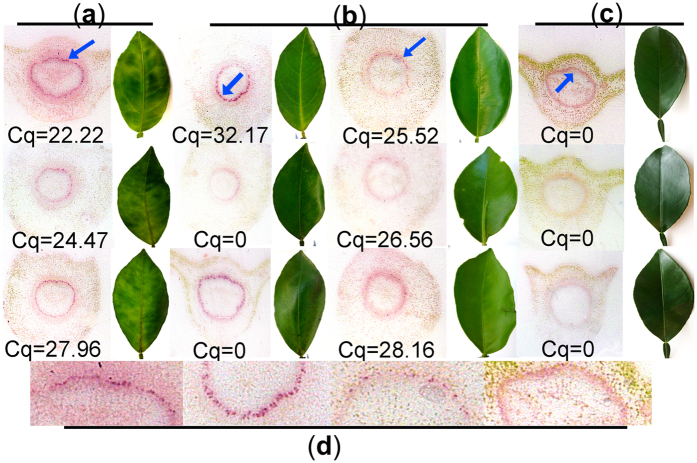
Detection of ‘*Ca*. Liberibacter asiaticus’ by tissue printing of symptomatic and asymptomatic sweet orange leaves collected from Florida citrus groves. Cq values are from qPCR assays performed on the same samples that were printed. Three leaves with blotchy mottle symptoms are on the left (**a**); and six leaves from the same tree but without symptoms are in the center (**b**); 3 leaves from a healthy tree on the right (**c**). Panel d contains higher magnification images of the tissue prints from the samples in the top row to illustrate the strict localization of the specific color reaction in phloem vessels.

To test the efficiency of the tissue print assay, 50 symptomatic and 50 asymptomatic leaf samples were collected in Florida from trees with symptoms of HLB and tested with both the tissue print and qPCR. Tissue prints from symptomatic petioles produced clear positive results for both tissue prints and qPCR (Fig. 5a). Tissue prints from asymptomatic leaf samples also produced clear positive reactions, even when the qPCR result was negative in some cases (Fig. 5b). In samples from known healthy controls, no purple colored spots localized in phloem cells or amplification products were observed (Fig. 5c), though a diffuse purple ring, without localization in the phloem cells, was seen in one of the samples (Fig. 5d). Among the symptomatic leaf samples tested and evaluated by 3 independent individuals, CaLas was detected in 72.7 ± 3.06% of the tissue prints and qPCR detected CaLas in 98.0 ± 0.58% of the samples (Table 1). Among asymptomatic leaf samples from infected trees, 82.8 ± 3.46% of tissue prints were positive, but only 64.0 ± 3.06% were positive by qPCR.

## Discussion

Serology is the method of choice used in plant pathology for detection of bacterial, viral and fungal pathogens. Specific polyclonal, monoclonal, or recombinant antibodies are available for many plant pathogens^32^,^33^,^34^,^35^. Monoclonal antibodies (Mabs) produced by hybridoma technology are highly specific against individual genera, species or isolates of plant pathogens or even for developmental stages of nematodes^36^. Mabs against CaLas were produced as early as in the 1980’s, but they were strain-specific and detected only the strain used for immunization but not another strain of CaLas^37^. Thus the Mabs were too specific for general diagnostic applications^38^ and the work was discontinued. Serological detection mainly relies on antibodies generated against unique antigens on the surfaces of plant pathogens. The detection thresholds of serologically-based assays vary significantly based on the type and quality of the antibody and the testing format^39^. OmpA was selected and used as antigen to produce polyclonal antibodies for serological detection of CaLas because it is present in high concentration on the surface of all cells of CaLas and the protein is generally conserved^40^. Polyclonal antibodies are able to recognize multiple epitopes within a single antigen. If some epitopes were shared between the strain of a pathogen used for immunization and the strain being diagnosed, cross-reactivity would be inevitable^41^. In the present study, we report the optimization of the working dilutions of both the anti-OmpA Pab rabbit antibody and the goat anti-rabbit secondary Pab. The optimal dilution for the anti-OmpA Pab was determined to be 1:5000. When the concentration of the anti OmpA Pab was too high, false positive results in the healthy controls were observed in the tissue prints. As the anti OmpA Pab was diluted more than 1:5000, it still produced good color signal in the phloem cells. Similar results were observed with the secondary goat anti-rabbit antibody. If diluted too much, no specific signal was detected, especially when the CaLas was at low levels. Taken together, the best concentration for anti-OmpA antibody was when diluted at 1:5000 and for the secondary goat anti-rabbit antibody when diluted at 1:50,000. We also describe the development of IC-PCR using the antibody and show how the results of qPCR, IC-PCR and tissue printing are correlated. In recent work, we have shown that the OmpA rabbit antibody is useful to study the distribution of CaLas in infected citrus trees^42^.

Tissue printing is one of the most widely used and simple techniques for cell-specific location of various macromolecules, such as proteins, enzymes, nucleic acids, or soluble metabolites in plant or animal cells^43^. The basic principle of tissue printing is that most of the cell contents, especially on the surface of a freshly cut tissue section, can be transferred to an adhesive or absorptive surface with little or no diffusion, by simple contact^43^ with excellent preservation of anatomical detail. The technique was used with nitrocellulose paper to localize extensin molecules in soybean tissue sections based on detection with silver or gold labeled antibodies^44^. Tissue printing has also been applied for the detection of RNA molecules^45^. Tissue printing has been widely applied to study the distribution and localization of viroids, viruses and bacteria in plants^45^,^46^. The tissue printing method also scales well and has the advantage of being easy to learn for inexperienced personnel who may take the samples in the field^47^.

Nucleic acid based probes were recently applied for the detection of CaLas in citrus plants and psyllids^48^ in tissue blots. These workers prepared tissue blots of nucleic acid extracts of infected plant material and then used a digoxygenin labeled probe and an anti-DIG labeled antibody to detect CaLas immobilized on the membranes. Anatomical resolution was lost in these tissue blots, unlike in our tissue prints. Others have spotted nucleic acid extracts from CaLas infected plants onto membranes and detected CaLas by PCR starting with the spotted nucleic acids on the membranes as template. The sensitivity and specificity of this method were as good as the qPCR assays used to detect the pathogen, avoided cumbersome purification of DNA prior to qPCR, and CaLas was detected in both symptomatic and asymptomatic leaf tissue^49^, with levels of detection similar to ours reported here. Our method has advantages of ease of implementation and the preservation of anatomical resolution, which increases the sensitivity of the assay, since a single infected phloem cells can be visualized. We also compared the results of tissue prints from symptomatic and asymptomatic leaf petioles with qPCR of DNA extracts prepared from the same petioles. We could detect the presence of CaLas in tissue prints from asymptomatic leaves from infected trees, even when the qPCR failed to detect the pathogen.

The anti-OmpA based tissue print technique also clearly showed details of the *in situ* distribution pattern of CaLas in different tissues of citrus^42^. As compared to tissue prints of sections of petioles, stems and seeds, the apparent concentration of CaLas in primary roots was lower. CaLas has been reported to colonize secondary roots prior to the development of visible foliar symptoms^28^, but such colonized secondary roots may die before visible symptoms occur, and thus would be unavailable for tissue printing. The concentration of viable CaLas in leaves was much higher than in primary roots^24^,^28^, consistent with this interpretation.

The tissue print assay requires viable cells, or at least cells with intact membranes, to present OmpA to the anti-OmpA Pab. Previous reports based on qPCR assays have demonstrated uneven distribution of CaLas cells in different tissues of infected sweet orange trees^24^,^25^. Our results provide a visual confirmation of this, and also provide previously unavailable anatomical resolution of the distribution of CaLas. The basis of organ or tissue preference in the colonization of citrus by CaLas remains unknown, and this technique could be applied to address this question.

Immune capture-PCR (IC-PCR) is a method that combines the advantages of antibody recognition of the pathogen with the amplification power of PCR. IC-PCR is a powerful method for detecting low quantities of protein antigens^50^,^51^. For the present study, in order to confirm that the anti-OmpA Pab can specifically capture CaLas from crude plant extracts, IC-PCR was performed following published methods^50^. The optimum detection of CaLas with IC-PCR using primer pair OI1/OI2c, was with the anti-OmpA Pab diluted at 1:200 and with plant extract volumes of 100 μl–200 μl. qPCR was used separately to estimate the number of CaLas genomes present in the plant extracts with a regression equation^52^. There was a general correlation between the concentration of CaLas estimated by qPCR in the crude extracts and the capture efficiency of anti-OmpA Pab based on the amount of amplification product produced following IC-PCR. When the Cq value was close to 25 (2.3 × 10^6^ copies of CaLas per gram of plant tissue), there was no visible amplification product following IC-PCR, suggesting that this is the practical limit of the technique. Immune capture of a PCR target is less expensive and easier to perform than the commonly used methods for the purification of DNA from infected plants prior to PCR. As noted above, IC-PCR and tissue printing would be expected to capture only viable cells with intact membranes using the anti OmpA Pab, in contrast to qPCR of total DNA recovered from infected plants, which would include DNA from both living and dead cells. Thus the results of the different assays are expected to be incompletely correlated.

Testing of field samples using the tissue print method was followed by qPCR on the same tissue sections. When plainly symptomatic leaf samples were tested, qPCR produced positive results on 98.0% of the samples, and only 73% of the same samples were positive by tissue printing. However, when asymptomatic leaf samples from the same trees were tested, the relative efficiencies of the two methods were reversed: qPCR produced positive results for only 64% of the samples, but tissue printing produced positive results for 83% of the samples. Thus the relative efficiencies of the two methods make them complementary and can be explained by the presence of both living and dead CaLas in plant tissues^29^. In symptomatic CaLas-infected citrus samples, the proportion of viable cells was only 17–31%. The DNA-based qPCR technique detects DNA from both living and dead cells^53^, whereas the antibody-based tissue printing method detects OmpA only in viable cells with intact membranes. Thus in symptomatic tissues, the concentration of DNA targets for qPCR would be higher than the concentration of antigenic targets for serological detection using this Pab. When asymptomatic leaf samples from known CaLas-positive trees were tested, higher Cq values indicated a lower concentration of CaLas cells, but the tissue printing indicated that most of CaLas were living and had intact membranes. When either symptomatic or asymptomatic samples were tested, both qPCR and tissue printing were about 80% efficient in detecting CaLas. Notably, the results were complementary so that when the two methods were combined, CaLas was detected in about 95% of the samples tested.

We have tried to use our anti-OmpA Pab in a standard DAS-ELISA format (unpublished data) but have been unsuccessful, despite our successful results with the Pab using the tissue printing format. We interpret this to mean that although the population of CaLas may have been high in individual phloem cells, in the plant tissues as a whole the population of viable CaLas was below the detection threshold for ELISA.

In conclusion, we have developed and validated a rapid and simple tissue print method for the serological detection of CaLas and demonstrated that it can be used to test field samples. Compared to qPCR, the current standard for detection of CaLas, it is easier to perform, much less expensive, readily scalable for many samples, equally reliable overall and is especially suited for detection of CaLas in asymptomatic tissues where their concentration is low. The tissue printing method described can also be used to rapidly and comprehensively detect viable CaLas in infected citrus trees. Our IC-PCR method also uses the anti-OmpA Pab to remove CaLas from crude plant extracts, which eliminates the need for DNA purification prior to PCR. The tissue printing assay is shown to be complementary to qPCR-based methods for detection of CaLas in infected trees, and when the methods are combined, both symptomatic and asymptomatic leaf samples from HLB affected trees can be confirmed to be positive for the pathogen at a 95% rate.

## Methods

### Plant Materials and Pathogens

Trees infected with ‘*Ca*. Liberibacter asiaticus’ were propagated by bud inoculation of either rough lemon (*Citrus jambhiri* Lush.) seedlings or sweet orange (*Citrus sinensis* [L.] Osbeck) propagated on rough lemon rootstocks. CaLas infected periwinkle (*Catharanthus roseus*) samples were propagated by branch graft inoculation to healthy periwinkle seedlings. All plants were grown in Metro Mix 510 potting mix in a greenhouse. The temperature of the greenhouse was maintained at 65–80F (18–27C) and ambient light was supplemented with high-pressure sodium vapor lighting on cloudy days and throughout the winter season to extend the photoperiod. Plants were watered as needed with water containing nitrogen/phosphorus/potassium (100/25/100 ppm), copper (2 ppm) and iron (6 ppm).

Field samples of ‘Valencia’ sweet orange (*Citrus sinensis* [L.] Osbeck) including symptomatic and asymptomatic leaf samples, root and fruit samples were obtained from groves in Florida.

### Preparation of anti-OmpA polyclonal antibodies

The rabbit polyclonal antibody (Pab) was prepared as described^32^.

### Tissue prints

HLB-affected and symptomatic leaves and corresponding healthy controls were collected from the greenhouse and kept on ice before preparing tissue prints. Tissue printing was performed according to a modified published protocol^47^. Sections approximately 2 mm thick were cut from the petioles of diseased or healthy tissue and pressed (10~15 seconds) onto nitrocellulose membranes (Whatman; 0.22 μm pore size). The membranes were air dried for 5 minutes at room temperature and then transferred to phosphate buffered saline with 0.05%Tween-20 (PBST) and washed two times (five minutes each) on a reciprocal shaker (80–100 rpm). The PBST was removed and replaced with SuperBlock (PBS) Blocking Buffer (Thermo Fisher Scientific) for initial blocking at room temperature for 2 h. The membranes were transferred to a standard blocking solution (PBST + 5% fat free skim milk) that contained the rabbit anti-OmpA Pab at different dilutions from 1:500, to 1:10,000 and incubated for 90 minutes at 37 C. Membranes were then washed three times with PBST for 10 min each. A secondary goat anti-rabbit polyclonal antibody conjugated with alkaline phosphatase was serially diluted from 1:10,000 to1:100,000, and incubated with the membranes for 1 h at 37 C. The tissue prints were washed three times with PBST for 10 min each before substrate was added (33 μl NBT + 16.5 μl BCIP; Sigma) in 5 ml of alkaline phosphate assay buffer. Incubation was stopped when purple color development could be seen. The tissue prints were photographed with a Carl Zeiss SteREO Discovery V20 light microscope equipped with a digital camera (AxioCamHR3).

The tissue prints prepared from leaves of field samples collected from HLB groves with and without symptoms were scored as positive or negative for CaLas by three individuals with no knowledge of the symptom status of the samples. For this purpose, a single purple colored spot in the phloem ring among the three replicates of a given sample resulted in the sample being declared positive. Leaves from healthy trees collected from the greenhouse were also tested in the same manner.

### Immune capture PCR (IC-PCR)

PCR tubes (0.2 ml) were pre-coated overnight at 4 C with 200 μl anti-OmpA Pab diluted (1:100, 1:200 and1:250) in ELISA coating buffer (0.1 M sodium carbonate, pH 9.5) and then washed three times with PBST to remove unbound Pabs. Extracts of CaLas-infected and healthy sweet orange and periwinkle plants were prepared by homogenizing midribs in ELISA coating buffer with 20% sucrose. Different volumes (20 μl, 50 μl, 100 μl and 200 μl) of these extracts were added by pipetting into the Pab coated 0.2 ml tubes in duplicate and also into 0.2 ml tubes that had not been pre coated with anti-OmpA Pab. Extracts of CaLas-infected plants in tubes with no Pab pre-coating as well as extracts of healthy plants in Pab coated tubes were both used as controls. The prepared samples were placed in an incubator at 37 C for 2 h, followed by two washes with PBST for 10 min each. DNA was extracted immediately from an equal volume of each starting extract for qPCR to determine the concentration of CaLas in each sample. The PCR reactions were carried out in 20 μl volumes that contained 1 × PCR reaction buffer, 2.5 mM MgCl_2_, 0.2 mM dNTP, 0.2 μM of forward and reverse primers (OI1/OI2c)^16^ and 1 U Platinum *Taq* DNA polymerase (Invitrogen, Frederick, MD). An extract from CaLas-infected citrus was used as the positive control. The thermal cycling program was set up in a PTC-200 (MJ Research)^27^.

### Extraction of DNA and qPCR Assay

After tissue prints were prepared, 6 petiole sections from each sample were collected for DNA extraction with the DNeasy kit (Qiagen, Valencia, CA). qPCR was performed using the plant mitochondrial cytochrome oxidase (COX) gene as an internal control^31^. The tissue print and qPCR assays were performed in triplicate.

## Additional Information

**How to cite this article:** Ding, F. *et al*. Immune Tissue Print and Immune Capture-PCR for Diagnosis and Detection of *Candidatus* Liberibacter Asiaticus. *Sci. Rep.* **7**, 46467; doi: 10.1038/srep46467 (2017).

**Publisher's note:** Springer Nature remains neutral with regard to jurisdictional claims in published maps and institutional affiliations.

## Supplementary Material

Supplementary Information

## Figures and Tables

**Table 1:** Detection of ‘*Ca*. Liberibacter asiaticus’ in field samples of sweet orange collected in Florida by tissue printing and qPCR.

Symptoms	Number Tested	Tissue Print (Positive %)	qPCR (Positive %	Combined (Positive %)
Blotchy mottle leaves	50	72.7% ± 3.06	98.0% ± 0.58	100%
Asymptomatic leaves	50	82.8% ± 3.46	64.0% ± 3.06	90.0% ± 1.63
Total	100	77.8% ± 10.7	81.0% ± 4.85	95.0% ± 1.33

The data are the mean percentage of samples declared positive by three independent evaluators ± the standard deviation of the means.
