# Supplementary material for: Immune Tissue Print and Immune Capture-PCR for Diagnosis and Detection of Candidatus Liberibacter Asiaticus
Source: Sci Rep. 2017 Apr 18;7:46467. doi: 10.1038/srep46467 (PMC5394477; doi:10.1038/srep46467)

Supplemental Materials

Immune Tissue Print and Immune Capture-PCR for Diagnosis and Detection of *Candidatus* Liberibacter Asiaticus

Fang Ding[1,2], Cristina Paul[2], Ron Brlansky[3] and John S. Hartung[2*]

Figure S1. Optimization of the dilution of primary rabbit anti-OmpA polyclonal antibody used for the detection and localization of '*Ca*. Liberibacter asiaticus' by tissue printing. 1:1000 (a); 1:2000 (b); 1:3000 (c);1:4000 (d); 1:5000 (e); 1:6000 (f); 1:7000 (g); 1:8000 (h); 1:9000 (i); 1:10,000 (j). Goat anti-rabbit polyclonal antibody diluted 1:50,000 was used throughout. CaLas strain B232 (Thailand) in sweet orange is on the left in each panel with healthy sweet orange on the right.

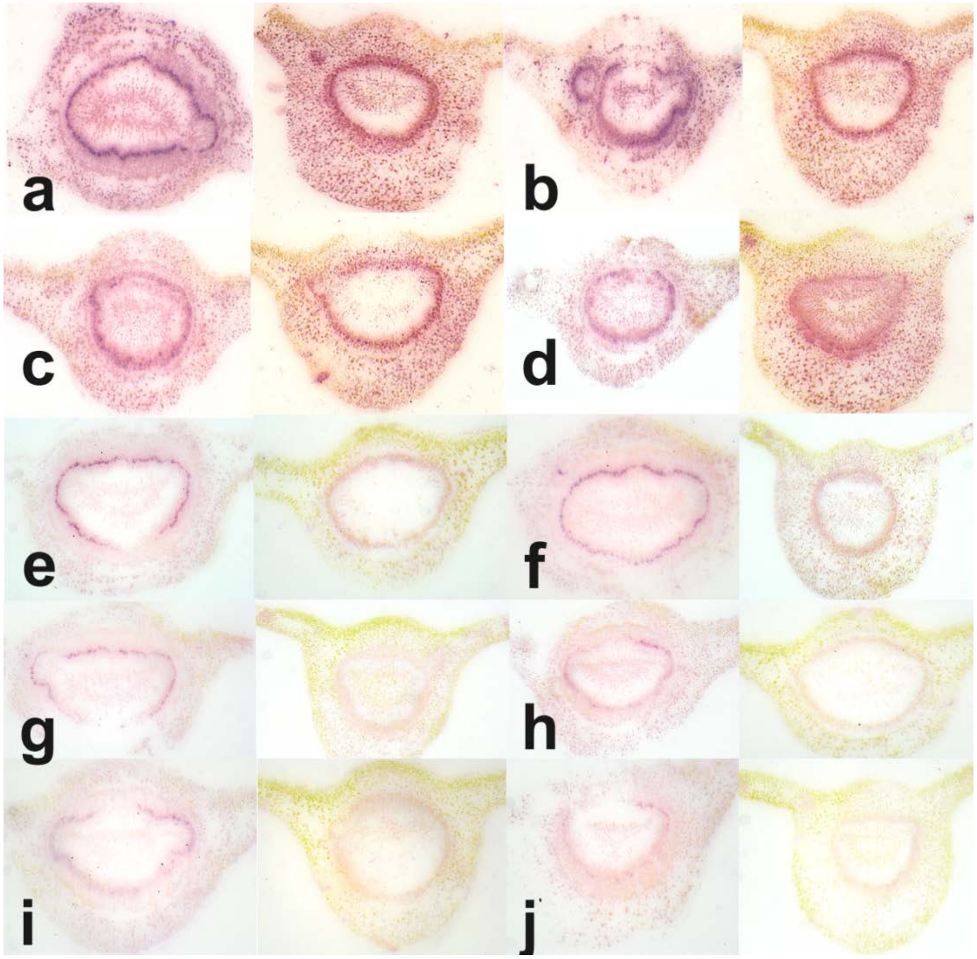

Figure S2. Optimization of the dilution of the secondary goat-anti-rabbit monoclonal antibody for the detection and localization of '*Ca*. Liberibacter asiaticus' by tissue print. 1:10,000 (a); 1:20,000 (b); 1:30,000 (c); 1:40,000 (d); 1:50,000 (e); 1:60,000 (f); 1:70,000 (g); 1:80,000 (h); 1:90,000 (i); 1:100,000 (j). Rabbit anti-OmpA polyclonal antibody diluted 1:5000 was used throughout. CaLas strain B232 (Thailand) in sweet orange on the left in each panel with healthy sweet orange on the right.

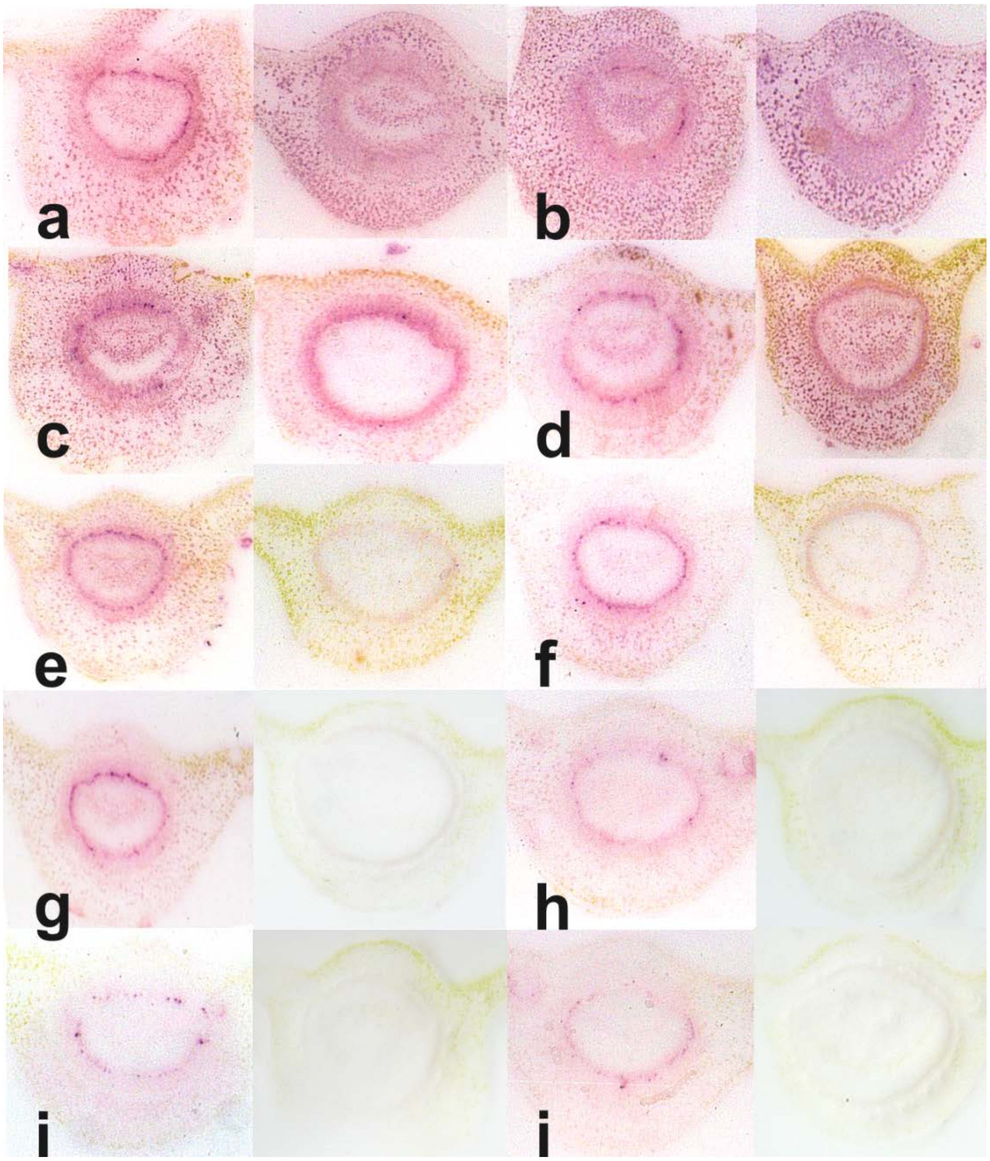

Supplement: Supplementary Information [file srep46467-s1.pdf]
